# THUMPD3 regulates alternative splicing of ECM transcripts in human lung cancer cells and promotes proliferation and migration

**DOI:** 10.1371/journal.pone.0314655

**Published:** 2024-12-10

**Authors:** Marie Klimontova, Han Zhang, Francisco Campos-Laborie, Natalie Webster, Byron Andrews, Kimberley Chung Kim Chung, Ryan Hili, Tony Kouzarides, Andrew J. Bannister

**Affiliations:** 1 The Gurdon Institute and Department of Pathology, University of Cambridge, Cambridge, United Kingdom; 2 STORM Therapeutics Ltd., Babraham Research Campus, Cambridge, United Kingdom; 3 Department of Chemistry and Centre for Research on Biomolecular Interactions, York University, Toronto, ON, Canada; 4 Milner Therapeutics Institute, University of Cambridge, Cambridge, United Kingdom; ShanghaiTech University, CHINA

## Abstract

RNA-modifying enzymes have recently garnered considerable attention due to their relevance in cancer biology, identifying them as potential targets for novel therapeutic intervention. THUMPD3 was recently identified as an RNA methyltransferase catalysing N^2^-methylguanosine (m^2^G) within certain tRNAs. In this study, we unveil a novel role for THUMPD3 in lung cancer cells. Depletion of the enzyme from lung cancer cells significantly impairs their fitness, negatively impacting key cellular processes such as proliferation and migration. Notably, exogenous expression of THUMPD3 in normal lung fibroblasts stimulates their proliferation rate. Additionally, transcriptome-wide analyses reveal that depletion of THUMPD3 from lung cancer cells induces substantial changes in the expression of cell surface proteins, including those comprising the extracellular matrix (ECM). We further demonstrate that THUMPD3 maintains expression of an extra-domain B (EDB) containing pro-tumour isoform of *Fibronectin-1* mRNA, encoding FN1, an important ECM protein. Crucially, depletion of THUMPD3 promotes an alternative splicing event that removes the EDB-encoding exon from *Fibronectin-1*. This is consistent with THUMPD3 depletion reducing cellular proliferation and migration. Moreover, depletion of THUMPD3 selectively and preferentially affects the alternative splicing of ECM and cell adhesion molecule encoding transcripts, as well as those encoding neurodevelopmental proteins. Overall, these findings highlight THUMPD3 as an important player in regulating cancer-relevant alternative splicing and they provide a rationale for further investigations into THUMPD3 as a candidate target in anti-cancer therapy.

## Introduction

Lung cancer stands as the foremost contributor to global cancer-related mortality [1]. Non-small cell lung cancer (NSCLC), comprising adenocarcinoma, squamous cell carcinoma, and large cell carcinoma, constitutes over 80% of diagnosed lung cancer cases [2]. The challenge in effectively addressing NSCLC lies in its often-advanced stage at diagnosis, with metastatic lesions commonly established by the time of presentation [3]. Moreover, resistance to drugs represents a significant factor contributing to the ineffectiveness of therapies in NSCLC, resulting in tumour recurrence and progression of the disease [4]. These inherent characteristics underscore the urgency for innovative approaches in the development of effective therapies for NSCLC.

The extracellular matrix (ECM) plays a pivotal role in regulating cell behaviour [5, 6], and its involvement in orchestrating tumour progression is increasingly being recognised across various cancer types [7]. Within lung cancer, the ECM encompasses a diverse array of proteins forming a dynamic network with structural and signalling roles, subject to continual remodelling [8]. Notably, in NSCLC, correlations between ECM composition and prognosis have been identified [9]. Indeed, certain ECM proteins, such as Tenascin C, are used as prognostic markers specifically in adenocarcinoma-type NSCLC [10, 11]. These findings underscore the significance of ECM dynamics in influencing the course of lung cancer.

Chemical modification to biological macromolecules—DNA, RNA, and proteins—play crucial roles in governing diverse cellular processes. While DNA and protein modifications have long been the focus of extensive research, RNA modifications, originally constrained by detection limitations, have only recently gained prominence with the advent of more sensitive techniques [12]. To date, over 150 RNA modifications have been identified across all domains of life [13, 14].

Three classes of proteins govern the intricate landscape of RNA modifications: ‘writers’ introduce modifications, ‘readers’ recognise these modifications, and ‘erasers’ remove them from RNA. This orchestrated interplay holds pivotal significance in numerous cellular processes [15]. Remarkably, many RNA-modifying enzymes are implicated in cancer, where they influence processes such as cell proliferation, invasion, migration, and contribute to cellular metabolism and drug resistance [16]. The significant linkage of RNA enzymes to various cancers has spurred focused efforts in developing inhibitors of the relevant enzymes. Notably, the development of an inhibitor for m^6^A methyltransferase, METTL3, has progressed to Phase 1 clinical trials for advanced malignancies (NCT05584111).

N^2^-methylguanosine (m^2^G), identified in tRNAs and rRNAs across species, plays crucial roles in maintaining structural integrity. In tRNAs, m^2^G is vital for structural fidelity, preventing aberrant conformations [17]. In rRNAs, m^2^G contributes to structural stabilisation, particularly at functionally significant sites [18]. Nevertheless, despite these insights, the role of m^2^G in broader RNA biology remains largely unexplored. Until recently, the enzymes catalysing m^2^G in higher eukaryotes were unknown. However, during the course of our study, three human proteins—THUMPD2, THUMPD3, and TRMT11—were identified as m^2^G RNA methyltransferases [19–21]. Initially, THUMPD3, together with the activator protein TRMT112, was found to deposit m^2^G at position 6 of specific human cytoplasmic tRNAs, both *in vitro* and *in vivo* [19]. Subsequently, it was demonstrated that THUMPD2 and TRMT11, each in a complex with TRMT112, also function as m^2^G methyltransferases [20]. TRMT11-TRMT112 specifically catalyses the formation of m^2^G at position 10 of certain tRNAs. Interestingly, while the THUMPD2-TRMT112 does not exhibit significant methylation activity towards tRNAs *in vitro*, it interacts with *U6* snRNA and is responsible for the methylation of the G72 nucleoside in *U6* snRNA [20, 21].

In this study, we have employed a range of cellular assays to explore the role of THUMPD3, a human m^2^G methyltransferase, in lung cancer cell biology, specifically NSCLC. We show that THUMPD3 regulates crucial aspects of lung cancer pathogenesis, including cell proliferation and migration. Moreover, whole-transcriptome analysis underlines the extensive impact of THUMPD3 depletion, showcasing its role in the dysregulation of multiple pathways in lung cancer cells. Notably, THUMPD3 depletion leads to significant effects on the levels of ECM transcripts and their encoded proteins. Collectively, our findings highlight that THUMPD3 plays a pivotal role in lung cancer maintenance by influencing ECM expression. We provide evidence for a novel mechanism in which THUMPD3 affects the alternative splicing of cancer relevant and ECM enriched mRNAs, such as *Fibronectin-1* mRNA, thereby promoting a molecular environment permissive to lung cancer progression. Overall, our work highlights THUMPD3 as a potential therapeutic target and it begins to unravel its intricate involvement in lung cancer biology.

## Results

### THUMPD3 regulates m^2^G in lung cancer cells

At the outset of this work, no human m^2^G RNA methyltransferases had been identified. Therefore, to expand the repertoire of human RNA methyltransferases and to explore the roles of the enzymes and the relevant modification in human cells, we sought to identify human m^2^G RNA methyltransferases.

**Fig 1:**
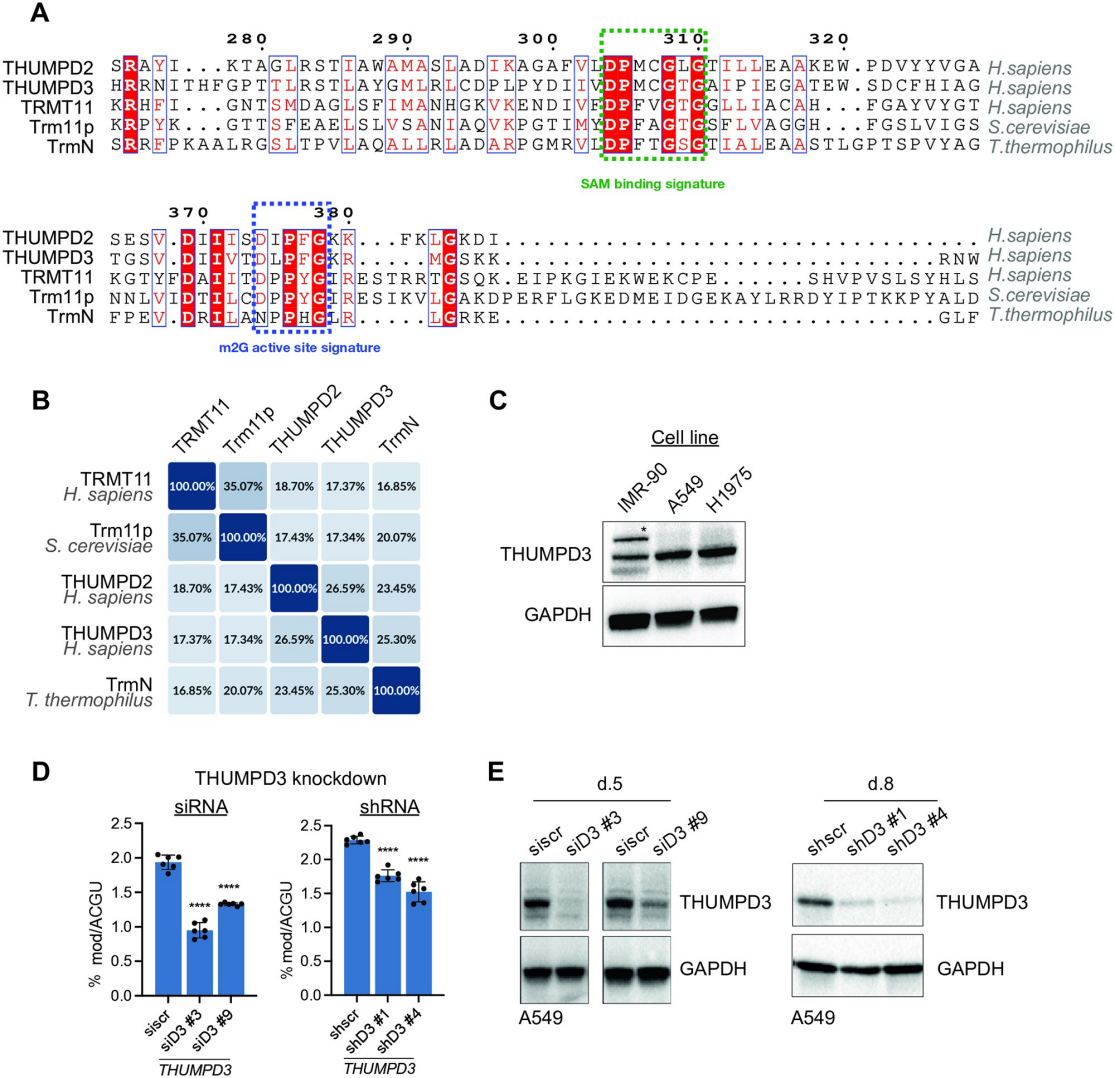
THUMPD3 is an m^2^G methyltransferase with a potential role in lung cancer. **(A)** Alignment of THUMPD2, THUMPD3, and TRMT11 to the known m^2^G methyltransferases like yeast Trm11p and bacterial TrmN. The predicted m^2^G active site and the SAM binding site are highlighted. The red colour signifies the conservation of specific amino acids across all species. **(B)** Diagram illustrating the percentage sequence similarity between the five m^2^G methyltransferases, as indicated. **(C)** Western blotting analysis of THUMPD3 levels in IMR-90 (normal lung fibroblasts), H1975 and A549 cells (lung adenocarcinoma) (top panel; left). The membrane was re-probed with an anti-GAPDH antibody as a loading control (bottom panel; left). The asterisk indicates a cross-reaction band. Quantification of THUMPD3 signal intensities normalised to GAPDH (graph; right). **(D)** MS analysis of RNA modifications in A549 cells following siRNA and shRNA mediated THUMPD3 depletion. For siRNA (left panel), cells were collected 5 days post transfection. For shRNA (right graph), cells were collected 8 days post induction of the shRNA. m^2^G abundance was measured using RNA MS. Each modification is presented as a percentage of ACGU. Statistical analysis was performed using One-Way ANOVA corrected for multiple comparisons using the Bonferroni method (Alpha: 0.05); ns—P > 0.05, *—P ≤ 0.05, **—P ≤ 0.01, ***—P ≤ 0.001, ****—P ≤ 0.0001. Error bars represent the mean ± standard deviation (SD) of 6 independent replicates. **(E)** Western blotting analysis upon THUMPD3 knockdown described in D. Anti-GAPDH antibody was used as a loading control.

To this end, we performed homology comparisons to known m^2^G RNA methyltransferases of other species, and we identified human THUMPD2, THUMPD3 and TRMT11 as potential m^2^G RNA methyltransferases (Fig 1A and 1B). This highlighted TRMT11, THUMPD2 and THUMPD3 as prime candidates for being human m^2^G RNA methyltransferase. Indeed, during the course of our study, all three human proteins were confirmed as m^2^G RNA methyltransferases [19–21]. Of these, we also identified that THUMPD3 is more highly expressed (more than 2-fold) in lung adenocarcinoma (A549 and H1975) cells than in normal IMR-90 lung fibroblasts (Fig 1C). Given this elevated expression, we decided to further explore THUMPD3 in lung tumourigenesis.

In initial analyses, siRNA and shRNA approaches were employed to deplete THUMPD3 from human lung adenocarcinoma cells (A549). The use of two distinct methods to target THUMPD3 minimised the risk of following off-target effects. As previous studies had implicated THUMPD3 in the methylation of tRNA species [19, 20], we first confirmed that the depletion of THUMPD3 from A549 cells reduced m^2^G in tRNAs. We therefore performed RNA MS analysis on tRNA isolated from control and THUMPD3 depleted A549 cells. As expected, the results confirmed a significant decrease of m^2^G in the small (≤200 nucleotides) RNA fraction following either siRNA or shRNA mediated THUMPD3 depletion (Fig 1D and 1E). Collectively, these findings indicate that THUMPD3 expression is elevated in lung cancer cells where it functions as an m^2^G tRNA methyltransferase.

### THUMPD3 impairs cell proliferation and migration of lung cancer cells

**Fig 2:**
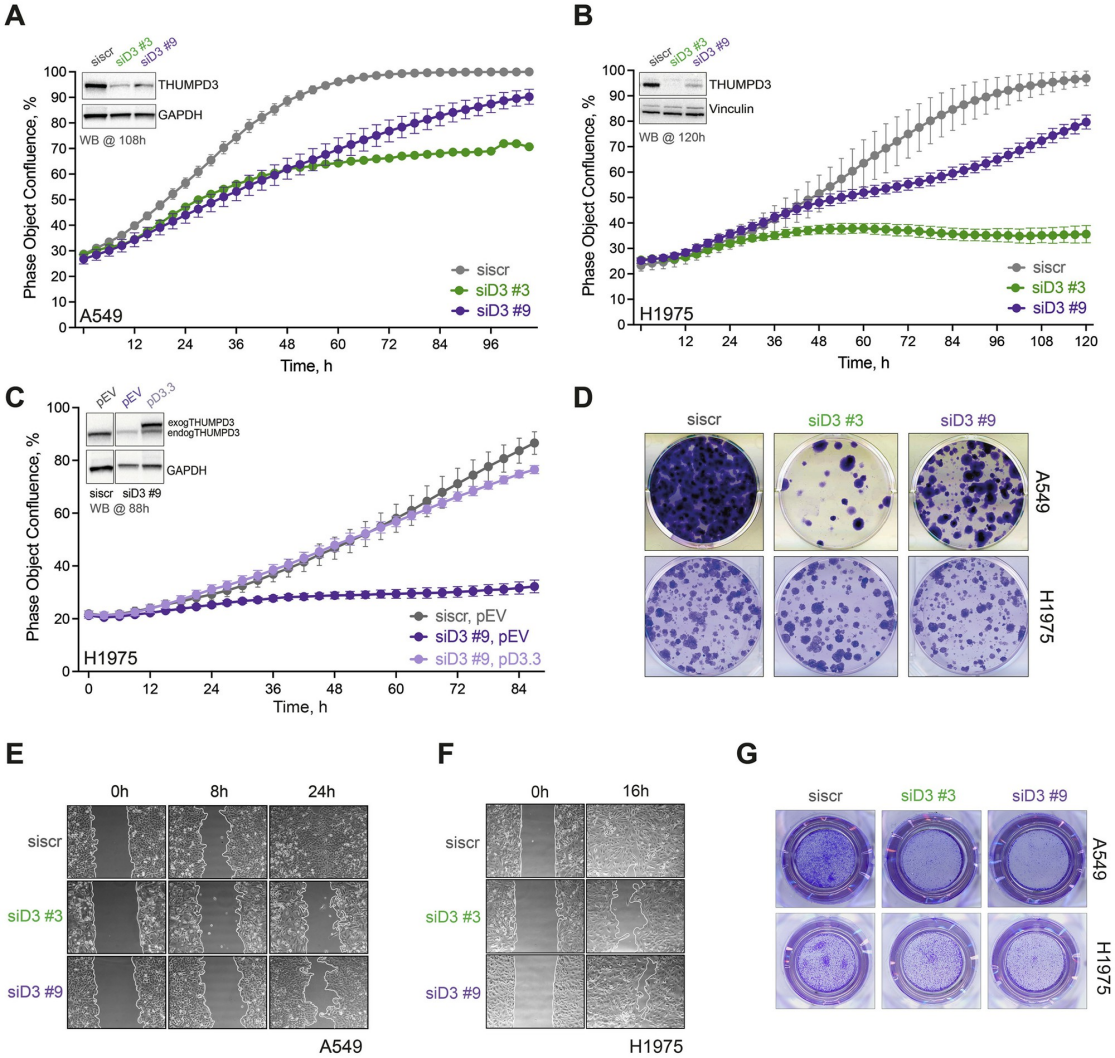
THUMPD3 depletion has negative impacts on lung cancer progression. **(A, B)** Live-cell imaging analysis of cell proliferation in A549 and H1975 cells upon THUMPD3 depletion via two independent siRNAs, as indicated. Data are represented as the mean of duplicates ± SD. Western blotting was performed upon completion of live-cell imaging and confirmed THUMPD3 depletion upon siRNA treatment (inset in graph). GAPDH or vinculin were used as a loading control, as indicated. **(C)** Rescue of proliferation defect in H1975 cells induced by THUMPD3 depletion by expression of exogenous THUMPD3 (pD3.3) but not by the empty vector control (pEV). Data are represented as the mean of duplicates ± SD. Western blotting (inset in graph) shows THUMPD3 levels following live-cell imaging. GAPDH was used as a loading control; endogTHUMPD3 and exogTHUMPD3—endogenous and exogenous THUMPD3, respectively. **(D)** Colony formation assay in A549 and H1975 cells upon THUMPD3 depletion using indicated siRNAs. Crystal violet staining was performed 2 weeks after colony formation. **(E, F)** Wound healing assay in A549 and H1975 cells upon THUMPD3 depletion with siRNAs as indicated. Wound closure (as a percentage of cell-free area) was determined; this reflects the width of the wound region (scratch) at a given time relative to the width at time zero when the scratch was created. **(G)** Transwell assay in A549 and H1975 cells upon THUMPD3 depletion using the indicated siRNAs.

To determine whether THUMPD3 is required for lung cancer cell growth, we adopted an siRNA approach to deplete the protein from two NSCLC cell lines, A549 and H1975. Live-cell imagining confirmed that depletion of the enzyme from either lung adenocarcinoma cell line hindered their proliferation (Fig 2A and 2B). In addition, THUMPD3 depletion from A549 cells via an independent shRNA approach also had a negative effect on cell proliferation (S1A–S1C Fig). Crucially, the growth defect was completely rescued by exogenous expression of non-targetable THUMPD3 (Fig 2C). Importantly though, depletion of THUMPD3 from normal, non-transformed lung fibroblasts did not significantly influence their proliferation rate (S1D Fig), suggesting a cancer cell specific dependence of THUMPD3.

To further investigate the role of THUMPD3 in lung cancer, the colony formation capacity of A549 and H1975 cells was assessed. THUMPD3 depletion from either lung cancer cell line impaired their colony formation (Fig 2D and S1E Fig). In addition, we discovered that overexpression of THUMPD3 not only enhanced lung cancer cell proliferation, but it also stimulated the proliferation rate of normal lung fibroblasts (S1F and S1G Fig). This highlights a potential oncogenic role for THUMPD3 in human lung cells.

We next investigated whether depletion of THUMPD3 impacts the metastatic potential of lung adenocarcinoma cells, a crucial hallmark of cancer [22]. For these and the following experiments we predominantly used an siRNA approach to streamline experiment numbers, but importantly all of our previous analyses indicated no obvious differences between siRNA or shRNA mediated THUMPD3 depletion. The effects of THUMPD3 depletion on cell migration were firstly evaluated using wound healing (scratch) assays. Wound healing was monitored for 24 hours, using light microscopy, after a scratch was introduced. Notably, decreased wound healing capacity was evident in THUMPD3 depleted cells compared to control cells, as early as 8 hours after scratch initiation (Fig 2E and 2F). This effect was recapitulated in cells where shRNA targeting of THUMPD3 was used (S1H Fig). To further investigate the effect of THUMPD3 depletion upon cell migration, a standard Transwell assay was conducted. Cells with siRNA mediated depletion of THUMPD3 displayed reduced migration compared to cells transfected with control siRNA (Fig 2G). Overall, these data support a role for THUMPD3 in lung cancer cell migration.

To further investigate whether the cell proliferation defect following THUMPD3 depletion is associated with apoptosis, we examined apoptotic cell death mediated by caspases. Caspases execute apoptosis by cleaving several essential proteins crucial for cellular function and survival, with PARP-1 being one of the most well-known caspase substrates. Caspase-mediated cleavage of PARP-1 is widely recognized as a hallmark of apoptosis [23]. To assess apoptosis following THUMPD3 depletion, we utilised an antibody that specifically detects the 24 kDa fragment of cleaved PARP-1. This indicated elevated levels of cleaved PARP-1 72 hours post-knockdown of THUMPD3, suggesting that THUMPD3 depletion induces apoptosis in (S1I Fig).

### THUMPD3 depletion induces significant transcriptome-wide changes in A549 cells

The above experiments demonstrate that depletion of THUMPD3 hampers the proliferation and migration of lung adenocarcinoma cells. However, the underlining mechanisms of these changes remain unknown. To gain deeper insights into pathways responsible for these observed phenotypes, we conducted a whole-transcriptome analysis on A549 cells using RNA-sequencing (RNA-seq) technology. THUMPD3 was depleted using two different approaches: siRNA and shRNA, with cells harvested 5- and 6-days post treatment initiation, respectively.

PCA analysis of RNA-seq data indicated strong sample and data quality, as evidenced by the clear distinction between untreated and treated samples, while untreated samples themselves showed no significant variation (S2A Fig). Additionally, replicates of the same experimental condition clustered closely together, further confirming the consistency and reliability of the data.

**Fig 3:**
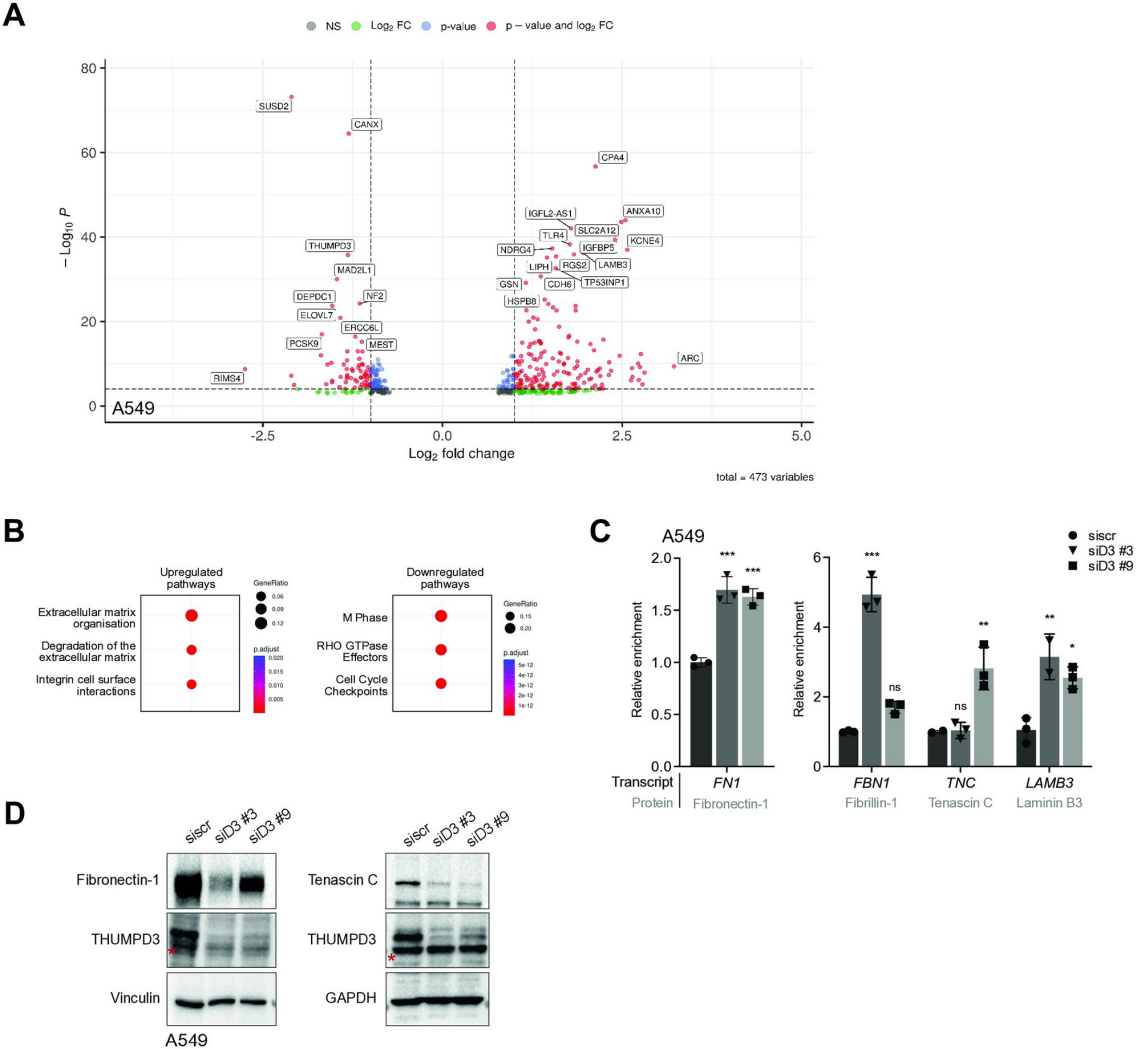
THUMPD3 depletion from A549 cells induces transcriptome-wide changes including deregulation of ECM transcripts. **(A)** Volcano plot representing global gene expression changes upon THUMPD3 depletion from A549 cells. The data show transcripts identified in both siRNA and shRNA mediated THUMPD3 knockdown analyses. The log2 fold change is plotted on the x-axis against -log10 of adjusted p-value plotted on the y-axis. Each point represents a single gene transcript. A single gene transcript with a significant fold change or p-value is represented in green or blue, respectively. When both parameters are significant for a gene, the corresponding dot is displayed in red. Significance cut-off for log2 fold change is 0.585 and for adjusted p-value is 0.05. **(B)** Reactome pathway analysis of A549 cells upon THUMPD3 depletion. Reactome enrichment statistics for top up- and downregulated pathways are ranked according to GeneRatio. The GeneRatio is defined as the number of genes up or downregulated associated with the given reactome pathway over the total number of up or downregulated genes. The dots are coloured according to adjusted p-value ranging from red (significant) to blue (less significant). Virus infection related pathways were removed for the simplification. **(C)** Validation of changes in abundance of specific upregulated transcripts in A549 cells upon THUMPD3 depletion induced by siRNAs, as indicated. Cells were collected 5 days post transfection. Transcript levels were measured and validated by qPCR analysis. The graph depicts relative enrichment normalised to GAPDH levels of upregulated transcripts, as indicated. Statistical analysis was performed using One-Way ANOVA corrected for multiple comparisons using the Bonferroni method (Alpha: 0.05); ns—P > 0.05, *—P ≤ 0.05, **—P ≤ 0.01, ***—P ≤ 0.001, ****—P ≤ 0.0001. Error bars represent the mean ± SD of 3 independent replicates. **(D)** Western blotting depicts reduced ECM protein levels induced by siRNA mediated THUMPD3 depletion (5 days post siRNA transfection). Anti-Fibronectin-1 and anti-Tenascin C antibodies were used, as indicated. Membranes were re-probed with anti-THUMPD3 antibodies. GAPDH and vinculin levels were used as loading controls, as indicated. The asterisk indicates a cross-reaction band.

Differential expression analysis identified a total of 284 transcripts that exhibited significant upregulation in both targeting approaches, while 189 gene transcripts were significantly downregulated in both approaches (Fig 3A, S2E–S2G Fig and S1 Table). Reassuringly, *THUMPD3* was one of the most downregulated transcripts, confirming efficient mRNA targeting and depletion.

We then conducted Reactome enrichment analysis to gain insights into biological processes influenced by THUMPD3 depletion (Fig 3B, S2B and S2C Fig). One of the most upregulated pathways was associated with extracellular matrix (ECM) organisation. Amongst downregulated pathways, key processes such as cell cycle regulation were identified. Overall, these findings establish an important role for THUMPD3 in various biological pathways likely critical for the efficient propagation and survival of lung cancer cells.

Real-time quantitative polymerase chain reaction (qPCR) validation of four transcripts encoding ECM proteins (Fibrillin-1, Fibronectin-1, Tenascin C and Laminin B3) confirmed they were significantly upregulated upon depletion of THUMPD3 (Fig 3C). Surprisingly though, when we assessed the levels of the 3 ECM proteins encoded by the upregulated transcripts, we found that they were significantly reduced following THUMPD3 depletion (Fig 3D and S2D Fig). This suggests that despite elevated ECM transcript levels in THUMPD3 depleted lung cancer cells, either their translation or the stability of the relevant encoded proteins was impaired. Additionally, it is well established that cells tend to downregulate their ECM proteins upon loosing cancerous properties [24], as they do with THUMPD3. An intriguing question remains as to whether the observed reduction in ECM protein levels is a direct or indirect consequence of THUMPD3 depletion. Ultimately though, the changes we see in ECM proteins following THUMPD3 depletion likely underpin, at least in part, the impaired cellular migration phenotype in THUMPD3 depleted lung cancer cells.

### THUMPD3 depletion affects alternative splicing

**Fig 4:**
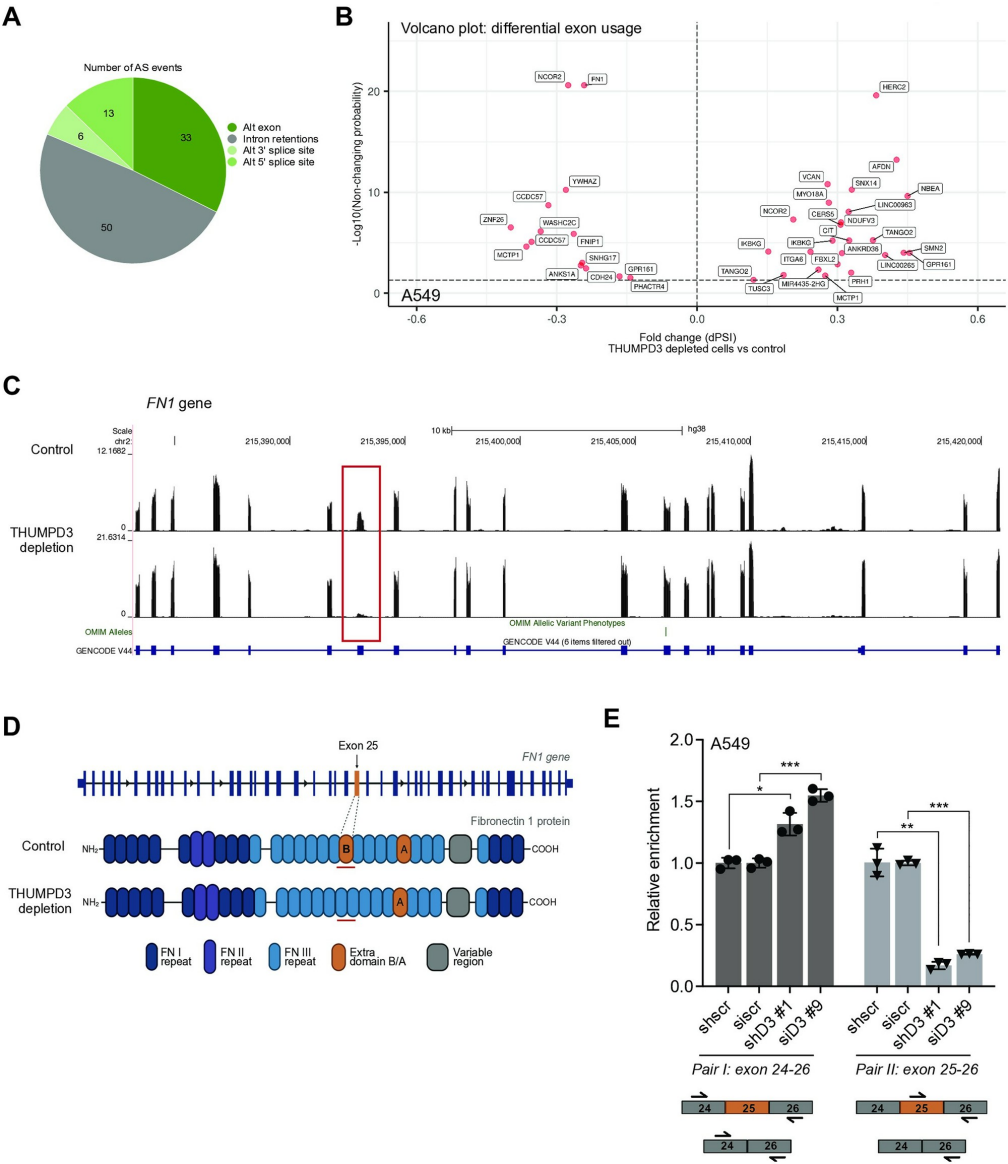
THUMPD3 depletion results in changes in alternative splicing of Fibronectin-1 transcript. **(A)** Overview of differential splicing analysis. In total 137 (102 unique) transcripts were identified. The bar chart represents the distribution of main AS events with an increased probability threshold of 0.95; Alt—alternative. **(B)** Volcano plot of differential splicing events: Alternative exon usage. The fold change of dPSI is plotted on the x-axis against -log10 of non-changing probability plotted on the y-axis. Each point represents a single transcript. **(C)** UCSC genome browser snapshot highlighting a reduction in usage of exon 25 (red box) in *FN1* (Fibronectin-1) upon THUMPD3 depletion in comparison to control cell RNA. **(D)** A cartoon structure of *FN1* gene and Fibronectin-1 protein (created in Biorender). **(E)** qPCR validation of exon 25 exclusion upon THUMPD3 depletion. For siRNA, cells were collected 5 days post transfection. For shRNA, cells were collected 8 days post doxycycline induction. Transcripts changes were measured by qPCR analysis. The graph depicts relative enrichment normalised to GAPDH levels and overall levels of *FN1* (primers designed to region outside of exon 25). Statistical analysis was performed using unpaired multiple t test corrected for the comparisons using the Bonferroni-Dunn method (Alpha: 0.05); ns—P > 0.05, *—P ≤ 0.05, **—P ≤ 0.01, ***—P ≤ 0.001, ****—P ≤ 0.0001. Error bars represent the mean ± SD of 3 independent replicates. A schematic illustrates annealing sites of primers with respect to exon 25 (bottom).

Given the recently identified link between THUMPD2 and RNA splicing [20, 21], we explored whether THUMPD3 was also involved in this process. While RNA-seq can be used to evaluate overall steady-state transcript levels, the resulting data can also be interrogated for changes in the relative abundance of gene isoforms, with alternative splicing (AS) being one main cause. Using stringent criteria, we evaluated our dataset for potential AS events induced by THUMPD3 depletion. In total, we identified 137 alternatively spliced events (S2 Table). In terms of AS event classification, intron retention emerged as the most prevalent (40%), followed by alternative exon usage (25%; Fig 4A). We generated volcano plots for the primary AS event types: alternative exon usage, intron retention, alternative 3’ and 5’ splice sites (Fig 4B and S3A–S3C Fig). Despite intron retention being the predominant alternative splicing event type detected, a review of the literature revealed no clear connection between the identified targets and THUMPD3. Additionally, we found no obvious links between these transcripts and the phenotypic changes observed in lung cancer cells following THUMPD3 depletion. In contrast, however, transcripts exhibiting alternative exon usage events were enriched in mRNAs associated with ECM and cell adhesion molecules (CAMs; S3D Fig, Table 1). In addition, substantial number of identified targets had some links to neurodevelopment (Table 2).

**Table 1:** THUMPD3 regulates alternative splicing of ECM and CAMs transcripts.

Gene ID	Name	Functional group	Citations^a^
*FN1* *	Fibronectin-1	ECM and CAMs	[27, 28]
*VCAN*	Versican	ECM and CAMs	[29]
*CDH24* *	Cadherin-24	ECM and CAMs	[30, 31]
*ITGA6*	Integrin alpha-6	ECM and CAMs	[32]
*AFDN*	Afadin	ECM and CAMs	[33]
*LINC00265*	*LINC00265*	Regulators of ECM and CAMs	[34]
*LINC00963*	*LINC00963*	Regulators of ECM and CAMs	[35]
*MYO18A*	Myosin XVIIIA	Regulators of ECM and CAMs	[36]
*PHACTR4* *	Phosphatase and actin regulator 4	Regulators of ECM and CAMs	[37]
*FNIP1*	Folliculin-interacting protein 1	Potential links to ECM and CAMs	[38]
*IKBKG*	Inhibitor of nuclear factor kappa-B kinase subunit gamma	Potential links to ECM and CAMs	[39]

ECM, extra cellular matrix; CAM, cell adhesion molecule.

*Also exhibit links to neurodevelopment.

^a^ Citations are illustrative and not exhaustive.

**Table 2:** THUMPD3 regulates alternative splicing of neurodevelopmental transcripts.

Gene ID	Name	Citations^a^
*ANKS1A*	Ankyrin repeat and sterile alpha motif domain containing 1A	[40]
*ANKRD36*	Ankyrin Repeat Domain 3	[41]
*CIT*	Citron Rho-interacting kinase	[42]
*NBEA*	Neurobeachin	[43]
*SMN2*	Survival motor neuron 1	[44]
*SNX14*	Sorting Nexin 14	[45]
*TANGO2*	Transport and Golgi organisation 2	[46]
*TUSC3*	Tumour suppressor candidate 3	[47]
*YWHAZ*	Tyrosine 3-Monooxygenase/Tryptophan 5-Monooxygenase Activation Protein Zeta	[48]

List of genes that have demonstrated links to neurodevelopment.

^a^ Citations are illustrative and not exhaustive.

Interestingly, one of the most statistically significant alternative exon usage events upon THUMPD3 depletion occurred within *FN1* mRNA (encoding Fibronectin-1), one of the upregulated ECM-related transcripts (Fig 4A and S1 Table). THUMPD3 depletion induced a highly selective decrease in usage of exon 25 within *FN1* (Fig 4C). Strikingly, exon 25 encodes a unique domain known as extra domain B (Fig 4D), which is associated with cancer-related functions [25, 26]. Importantly, qPCR analysis confirmed that THUMPD3 depletion results in exon 25 loss (Fig 4E).

## Discussion

Employing a diverse and orthogonal set of techniques, we show that targeted depletion of THUMPD3 from lung cancer cells impairs their growth. Depletion of the RNA methyltransferase affects expression of ECM proteins and critical cellular processes, including cell cycle regulation. Notably, we discovered that the expression and alternative splicing of one specific RNA encoding Fibronectin-1, an ECM protein, was significantly affected, and this correlated with changes in levels of Fibronectin-1.

Alternative splicing serves as a highly effective mechanism for regulating genomic diversity. It is also one of the most dysregulated pathways in cancer [49]. Notably, AS of ECM proteins plays a crucial role in stromal activation and disease progression [50]. Our findings identify a shift in *FN1* isoform expression upon THUMPD3 depletion that favours production of an *FN1* variant lacking the pro-angiogenic Extra Domain B (EDB), which has been previously associated with cancer progression [24, 25, 51]. This is an intriguing finding, and suggests that THUMPD3 contributes to cancer progression, at least in part, by maintaining inclusion of a cancer promoting exon in *FN1*.

The ability of tumour cells to metastasise is one of the hallmarks of cancer [22]. THUMPD3 depletion from lung cancer cells significantly impaired their migration capabilities. The ECM plays a pivotal role in regulation of this process [52], therefore changes such as the one we identified in *FN1* AS may help explain THUMPD3’s involvement. Furthermore, our finding that a third of transcripts identified as being subject to alternative exon usage following THUMPD3 depletion are strongly associated with ECM or CAMs, suggests a specific and preferential involvement of THUMPD3 in regulating the AS of critical migration associated proteins. Interestingly, a significant and overlapping proportion of transcripts with alternatively spliced exons also show links to neurodevelopment. Previous research has indicated that the *THUMPD3* gene is located in the 3p25.3 region, and interstitial deletions in this region lead to the 3p-syndrome, causing intellectual disability in patients [53]. However, more work is needed here to further explore these intriguing connections.

Notably, numerous mRNAs encoding ECM proteins, including Fibronectin-1, are significantly upregulated by depletion of THUMPD3. However, there is a dichotomy between changes in mRNA levels versus changes in protein levels; for all ECM factors analysed, their mRNAs increased in abundance whereas their protein levels decreased. One possible explanation for the observed reduction in protein levels following THUMPD3 depletion is that loss of the tRNA methyltransferase leads to the inhibition of global translation. However, previous reports present conflicting findings in this area. For instance, Yang *et al.* reported that while THUMPD3 depletion did affect polysome numbers, the impact was quite subtle [19]. Additionally, Wang *et al.* did not observe a significant reduction in polysome formation [20]. Nevertheless, they did find that THUMPD3 depletion led to an accumulation of monosomes and slight reduction in protein synthesis [20]. Clearly, further research is required to fully understand the role of THUMPD3 in the suppression of global translation. In any case, our loading controls will have controlled for a global downregulation of translation. The fact that we see significant downregulation of ECM proteins even after loading control correction, signifies a robust downregulation of their protein levels beyond the reduced levels expected from just suppression of global translation. Thus, the elevated levels of ECM and CAMs transcripts possibly stem from compensatory mechanisms attempting to overcome the reduced global translation.

The effects described above result from depletion of THUMPD3 from cells. This consequently leads to a marked reduction of m^2^G in RNA. However, it remains uncertain whether the effects we observed are solely attributable to THUMPD3’s role as an m^2^G methyltransferase or whether THUMPD3 possesses additional functions beyond its methyltransferase activity. This will be important to address in the future. Moreover, our findings raise a significant question concerning how THUMPD3 promotes one specific RNA splicing isoform over another. It is known that the related RNA methyltransferase THUMPD2 methylates *U*-snRNAs [20, 21], which are involved in splicing [54]. It is possible that THUMPD3 somehow interacts with the THUMPD2 enzyme to modulate this process. Alternatively, but not mutually exclusively, THUMPD3 might directly methylate one or more *U*-snRNAs, thereby regulating their function, or it could potentially methylate mRNAs themselves, influencing splicing. In addition, our RNA-seq data indicate that some *U*-snRNAs are downregulated following THUMPD3 depletion. However, the exact mechanism by which THUMPD3 could be involved in alternative splicing remains unclear. Regardless of which scenario is at play in lung cancer cells, gaining a detailed mechanistic understanding will necessitate the development of more sensitive technologies to detect m^2^G in RNAs than those currently available.

An additional aspect worth considering is a potential role played by *THUMPD3-AS1*, a long lncRNA that partially overlaps with the *THUMPD3* gene. Intriguingly, previous research highlighted the involvement of *THUMPD3-AS1* in lung cancer cell proliferation [55]. Investigating the potential interplay between THUMPD3 and *THUMPD3-AS1* could yield significant insights. Notably, existing studies characterising the role of *THUMPD3-AS1* in cancer have not examined the consequential effects (if any) on THUMPD3 levels following *THUMPD3-AS1* depletion. It is conceivable that the observed impairment in proliferation upon THUMPD3-AS1 depletion could be attributable to alterations in THUMPD3 protein levels or vice versa. Additionally, the possibility of further interactions, such as THUMPD3-dependent methylation of *THUMPD3-AS1*, could be investigated.

Here, we have uncovered a pivotal role for THUMPD3 in promoting lung cancer cell proliferation and migration. We find that the enzyme plays a crucial role in the regulation of the ECM, including the AS of *Fibronectin-1*, favouring a pro-metastatic isoform. We posit that THUMPD3 contributes, at least in part, to the maintenance of lung cancer via these mechanisms. Furthermore, our experiments also revealed that overexpression of THUMPD3 increases proliferation of both lung cancer cells and normal lung fibroblasts. These findings indicate that THUMPD3 may function as a novel oncogene contributing to the development of lung cancer. This observation is consistent with the activity of other RNA methyltransferases, such as METTL3, which promotes various cancer types, including AML, glioblastoma, and colorectal cancer [56]. Importantly, we also found that depletion of THUMPD3 from normal, non-transformed human lung fibroblasts did not significantly influence their proliferation rate. This highlights a specific and significant role for THUMPD3 in the context of lung cancer cell growth and provides support and rationale for the initiation of a THUMPD3 drug discovery programme.

## Materials and methods

### Cell culture

Cell culture was carried out under sterile conditions in a standard laminar flow hood. Human cell lines were maintained in filter top flasks or culture dishes and cultured in respective growth media. 293T, A549 and IMR-90 cell lines were grown in DMEM supplemented with 1 x penicillin-streptomycin (pen-strep) antibiotics and 10% [v/v] foetal bovine serum (FBS). H1975 cells were grown in RPMI media supplemented with 1x pen-strep antibiotics and 10% [v/v] FBS. Cells were grown at 37°C, 5% [v/v] CO_2_ and were maintained at ≈ 80% viability. Cells were passaged every 3–5 days and seeded at 1 x 10^5^ live cells per ml. Cells were passaged using conventional cell culture techniques. The maximum number of passages was 20 before a new vial of cells was revived. Tests for mycoplasma contamination were carried out every month or when a new vial of cells was revived.

### Transient transfection with siRNA

ON-TARGETplus (Dharmacon) or FlexiTube (Qiagen) siRNAs were used for the knockdown experiments according to the manufacturer’s instructions. 0.5–3 x 10^5^ cells per well were used for transfections in 6-well plates. siRNA sequences are listed in S3 Table.

### Transfection with plasmids for protein overexpression

1 x 10^5^ cells per well were plated into 6-well plates. Cells were allowed to settle and attach overnight. Next morning, transfection was carried out using Lipofectamine^™^ 3000 Transfection Reagent (Thermo Fisher, L3000001) according to the manufacturer’s instructions.

### Lentiviral production and infection

The ‘all-in-one’ pLKO-Tet-On system was used to generate inducible shRNA mediated THUMPD3 knockdowns [57, 58]. 293T cells were seeded onto L-polylysine (1 *μ*g/ml) coated 10 cm dishes 24 hours prior to transfection in antibiotic-free DMEM media with 10% [v/v] FBS. Cells were 80% confluent on the day of transfection. To produce lentiviral particles, cells were transfected using 42 *μ*l FuGENE^®^ 4K Transfection Reagent (Promega, E5911) with 6 *μ*g of l pLKO-Tet-On constructs together with 3.5 *μ*g psPAX2 (Addgene, 12260) and 4 *μ*g pCMV-VSV-G (Addgene, 8454). 16 hours later, the media was exchanged for fresh media. 48 hours post-infection, virus particles were harvested and sterile filtered using 0.45 *μ*m syringe filters (Millipore). Aliquots of lentiviral supernatants were stored at -70°C. Target cells (A549) were then transduced with the virus with polybrene (8 *μ*g/ml) to increase the efficiency of transduction. 48 hours after infection transduced cells were selected for by treatment with puromycin (1 *μ*g/ml). To induce expression of shRNAs, 10–100 ng/ml doxycycline was added to the media and the cells were incubated for 4–8 days to allow adequate expression of the shRNA. Target sequences of shRNA are listed in S3 Table.

### Cell proliferation assays using Incucyte^®^ S3 live-cell imaging

0.5–2 x 10^5^ cells per well (A549, H1975 or IMR-90) were seeded in 6-well dishes and reverse transfected with 2.5 nM of control (siscr) and THUMPD3 (siD3 #3, 9) siRNAs. Next day the media was changed, and plates were placed into an Incucyte^®^ machine for live-cell imaging. The confluence of cells at time point zero was maintained at ∼ 20% to allow a proper curve to be formed. The algorithm is based on measuring cell proliferation using live-cell time-lapse imaging without labels using Classic Confluence Analysis.

### Development of stable cell lines

Stable cell lines were developed as described by Ebrahimi *et al.*, 2015 [59]. Essentially, the pCMV6-Entry (pEV) and pCMV6-THUMPD3 (pD3.3) (S3 Table) were linearised using BglII enzyme (NEB, R0144S) according to the manufacturer’s instructions. Following electrophoresis of the resulting digestion product in a 1% [w/v] agarose gel, the linear plasmid was purified using standard QIAquick Gel Extraction Kit (Qiagen, 28706X4). H1975 cells were transfected with 1 *μ*g of the purified linearised vector as described above. Next day culture medium was refreshed. 48 hours post transfection, the cells were treated with 400 *μ*g/ml of G418 antibiotic to select for transfected cells. Once control cells without plasmid died, the concentration of G418 was reduced to 175 *μ*g/ml. Successful overexpression of THUMPD3 in the relevant stable cell lines was confirmed by Western blotting with anti-THUMPD3 antibody. Cell lines with equivalent expression of endogenous and exogenous THUMPD3 were selected for further analyses, such as rescue experiments.

### THUMPD3 rescue experiment

H1975 cells stably harbouring control (pEV) or THUMPD3 (pD3.3) expression vectors were used. 3 x 10^4^ cells per well were seeded into a 12-well plate and reverse transfected with 2.5 nM of control (siscr) and THUMPD3 (siD3 #9) siRNAs. 24 hours later the media was exchanged, and the plate was placed into the Incucyte^®^ instrument to follow cell growth.

### Crystal violet staining

0.5 ml/well for 12-well or 1 ml/well for 6-well dish of Crystal violet (CV) solution (0.05% [w/v] Crystal violet, 20% [v/v] ethanol) were added to fix and stain cells. Cells were incubated in the solution for 5 minutes on a shaker (slow shake). Plates were washed by submerging in a 1 litre beaker with H_2_O, allowed to dry overnight and imaged on an Epson Perfection V800 Photo scanner.

### Colony formation assay

A549 (1000 cells/well) and H1975 (2000 cells/well) were seeded into 6-well plates in a total volume of 2 ml per well. Colonies were allowed to form over 10–14 days. Colonies were then fixed and stained with CV and imaged using an Epson Perfection V800 Photo scanner.

### Wound healing assay

A549 or H1975 cells were reverse transfected with 2.5 nM of control (siscr) and THUMPD3 (siD3 #3, 9) siRNAs. 72 hour later 2 x 10^4^ cells were seeded into ibidi chambers (Culture-Inserts, 2 wells/chambers; ibiTreat, 80206) and allowed to form a monolayer. 24 hours later the Culture-Inserts were removed (’scratch’ was introduced). The chamber was filled with 1 ml of medium. To ensure that images were taken at the same field, a line was drawn perpendicular to the scratch at the bottom of the imaged field. Each image was then taken at the same spot. Pictures were taken at 0–24 hours post scratch using a Leica EC3 digital camera. Images were analysed using Image J software. The area of the wound was measured using the Image J plugin—MRI wound healing [60].

### Transwell^®^ migration assay

Transwell^®^ inserts (8 *μ*m; Corning^®^, 354480/1) were used to assess the migration capacity of cells upon THUMPD3 depletion. 2 x 10^5^ cells (A549 or H1975) were reverse transfected with 2.5 nM of control (siscr) and THUMPD3 (siD3 #3, 9) siRNAs. After 72 hours, cells were serum starved for 24 hours. The following day, 800 *μ*l DMEM 10% [v/v] FBS were added to the bottom of the empty well (12-well plate). Transwell^®^ chambers were then added to the wells with sterile tweezers, ensuring no bubbles were introduced. The cells were washed, detached with 1x trypsin-EDTA or cell dissociation buffer enzyme-free PBS-based (Gibco, 13151–014) and neutralised in DMEM 2% [v/v] FBS. The cells were counted and prepared at a concentration of 1 x 10^5^ cells per ml in serum-free media. 300 ml of cell suspension (3 x 10^4^ cells) was added to each Transwell^®^ chamber. The plates were then incubated overnight at 37°C. 24 hours post-seeding, the Transwells^®^ were removed and washed by submerging in 1x PBS. The migrated cells were then fixed and stained using CV, and imaged on an Epson Perfection V800 Photo scanner.

### Immunoblotting

Protein samples were harvested in RIPA buffer (25 mM Tris-HCl pH 7.6, 150 mM NaCl, 1% [v/v] NP-40, 1% [w/v] sodium deoxycholate, 0.1% [w/v] SDS) supplemented with protease and phosphatase inhibitor tablets, followed by sonication in Bioruptor Pico, Diagenode. Lysate was cleared by centrifugation at 12,000 × g and quantified using a DC Protein Assay Kit (BIO-RAD). Samples were run on SurePAGE^™^ precast polyacrylamide gels and transferred to nitrocellulose membrane. Membranes were blocked in 5% [w/v] BSA (or powdered milk) in 1x TBS, 0.1% [v/v] Tween20 (TBS-T) and then incubated at 4°C with primary antibodies. Bound antibody was detected with ECL solution following the manufacturer’s instructions (Promega, W1015). The membrane was imaged via a Chemidoc^™^ imaging system (BioRad). Antibodies and reagents listed in S3 Table.

### RT-qPCR

RNeasy Mini Kit (QIAGEN, 74104) was used for RNA extraction. An additional step of DNase digestion was incorporated after the first wash following the RNase-Free DNase Set protocol (QIAGEN, 79254). To prepare cDNA, RNA was reverse transcribed using the SuperScript^™^ III Reverse Transcriptase (Invitrogen^™^, 8080044). qPCR analysis was performed using a StepOnePlus^™^ Real-Time PCR System (Applied Biosystems^™^, Thermo Fisher, 4376357) with Power SYBR Green Master Mix Kit (Applied Biosystems^™^, Thermo Fisher, A25741). Primers listed in S3 Table.

### Purification of small and large RNA fractions

RNA Clean & Concentrator kits (ZYMO RESEARCH, R1013 or R1017) were used to separate small (≤ 200nt) RNA fraction, following the manufacturer’s instructions. RNA concentration was determined using a Qubit^™^ RNA HS Assay Kit (Thermo Fisher, Q32855).

### Mass spectrometry analysis of RNA nucleoside m^2^G

Nucleosides were prepared from enzyme-processed RNA by enzymatic digestion, using a cocktail of Benzonase (Merck), Phosphodiesterase 1 (Merck), and Antarctic Phosphatase (New England Biolabs) as described previously [61]. The reactions were filtered using an Amicon 30kDa MWCO spin-column (Merck) to remove protein and the filtrate was mixed with a 2 x loading buffer containing 0.1% formic acid and an internal standard (13C-labeled uridine generated from 645672–1MG Merck KGaA, previously treated with Antarctic Phosphatase). The samples were loaded onto an ACQUITY UPLC HSS T3 Column, 100 Å, 1.8 *μ*m, 1 mm X 100 mm (Waters Corp., Milford, MA, USA) and resolved using a gradient of 2%–10% acetonitrile in 0.1% formic acid over 10 min. MS analysis was performed in positive ion mode on an Orbitrap QExactive HF (Thermo Fisher, Waltham, MA, USA) mass spectrometer. Standard dilutions of all experimental nucleosides were prepared and analysed in parallel. There were three technical replicates of each sample and the analytical processing was performed using XCalibur Software (Thermo Fisher).

### RNA-sequencing

Whole transcriptome analysis was performed using RNA-sequencing (RNA-seq) technology. Total RNA, depleted of rRNA, was used as an input material for RNA-seq. rRNA was removed using Ribo-Zero^®^ rRNA Removal Kit (Illumina) following the reference guide. RNA Clean & Concentrator-5 kit (ZYMO RESEARCH, R1013) was used to clean up the rRNA depleted RNA sample. RNA was eluted in 15 *μ*l nuclease-free water. The efficiency of rRNA removal was assessed using an RNA Screen Tape (Agilent, 5067–5576) analysed on a 4200 TapeStation System (Agilent, G2991BA). 13 *μ*l of rRNA depleted RNA sample were used for the preparation of the RNA-seq library. The NEXTFLEX^®^ Rapid Directional RNA- Seq Kit 2.0 protocol was followed to perform all the remaining steps for library construction steps.

### RNA-seq data analysis

Reads were aligned to the human reference genome (GRCh38.p14) using STAR (v2.7.10a), with –quantMode GeneCounts to quantify gene counts with the annotation source NCBI RefSeq reference transcriptome GTF file [62]. Processing steps including gene filtering, normalisation and subsequent differential gene expression analysis were performed using the DESeq2 (v1.38.3) package in R (v4.2.0) [63].

In the DESeq2 workflow, two experimental variables were considered for the experimental design. The first variable was ‘Type,’ representing the type of gene knockdown (shD3 #1, shD3 #4, siD3 #3). The second variable was ‘Condition,’ representing untreated versus treated samples. Three biological replicates were used for each condition. Differential analyses were performed on all types of knockdowns using the formula: ∼ Condition + Type + Condition:Type. Additionally, analyses were conducted on each knockdown type individually using the formula: ∼ Condition, where treated samples were compared to untreated samples (reference level). Adjusted p-values were calculated using the Benjamini-Hochberg method as implemented in DESeq2 [64]. Fold changes were shrunk using the ashr and apeglm methods as implemented in DESeq2 [65, 66].

Functional enrichment analyses, encompassing Gene Ontology (GO), Reactome and KEGG pathway analyses, were conducted using the clusterProfiler package (v4.7.1.3) in R [67]. The list of significantly differentially expressed genes from DESeq2 was used for these analyses.

### Differential splicing data analysis

Alternative splicing (AS) events between untreated and treated shD3 #1 samples, representing conditions without and with THUMPD3 depletion, respectively, were identified from aligned reads using MAJIQ (Modelling Alternative Junction Inclusion Quantification) (v2.4) software [69, 69] and its associated visualisation package VOILA (v2.4). Default parameters were used, including –threshold 0.2 and –changing-pvalue-threshold 0.05. The AS information of Local Splicing Variations (LSVs) was further parsed and classified into subtypes of AS events (such as exon skipping, alternative intron, 5’ alternative splice sites, 3’ alternative splice sites etc.) using the MAJIQ Modulizer program with stringent parameters: 1) maximum dPSI value ≥ 0.2, 2) p value for determining whether a LSV is changing ≤ 0.05, 3) probability that a LSV is changing ≥ 0.95.

## Supporting information

S1 Fig Further characterisation on THUMPD3’s role in lung cancer cells and lung fibroblasts. **(A)** Crystal violet staining of A549 cells with shRNA mediated THUMPD3 depletion (6 days post induction). **(B)** Effect of shRNA mediated THUMPD3 depletion on cell proliferation assessed by cell counting. The graph represents data from 3 replicates per condition. Statistical analysis was performed using One-Way ANOVA corrected for the comparisons using the Bonferroni method (Alpha: 0.05); ns—P > 0.05, *—P ≤ 0.05, **—P ≤ 0.01, ***—P ≤ 0.001, ****—P ≤ 0.0001. Error bars represent the mean ± SD of 6 independent replicates. **(C)** Western blotting representing reduction in THUMPD3 level upon shRNA induction. GAPDH was used as a loading control. **(D)** Live-cell imaging analysis of IMR-90 cell proliferation upon THUMPD3 depletion. Data are represented as the mean of duplicates ± SD. Western blotting was performed to validate THUMPD3 depletion upon siRNA treatment (inset in graph). GAPDH was used as a loading control. The asterisk indicates a cross-reaction band. **(E)** Quantification (manual) of differences in colony formation in H1975 cells upon THUMPD3 depletion. The bar chart represents percentage of formed colonies normalised to control (mean of 2 experiments ± SD). **(F)** Live-cell imaging analysis of H1975 cells stably harbouring control empty vector (pEV) or exogenous THUMPD3 expression vector (pD3.3) and transfected with control siRNA (siscr). Following analysis, THUMPD3 levels were assessed by Western blotting (insets in graph). GAPDH was used as a loading control. **(G)** Live-cell imaging analysis of IMR-90 cells expressing exogenous THUMPD3 (pD3). Following analysis, THUMPD3 levels were assessed by Western blotting (insets in graph). GAPDH was used as a loading control. **(H)** Wound healing assay upon shRNA mediated THUMPD3 depletion in A549 cells. Representative light-field images of wound healing at indicated time points. Migration fronts are highlighted by blue lines. **(I)** 200,000 A549 cells were reverse transfected with 2.5 nM of control (siscr) and THUMPD3 (siD3 #3, #9) siRNAs, as indicated. 72 hours later, cells were collected for Western blotting analysis with anti-cleaved PARP1 and anti-THUMPD3 antibodies, as indicated. GAPDH was used as a loading control.
(TIF)

S2 Fig Gene expression changes induced by THUMPD3 depletion. **(A)** PCA (principal component analysis) based on gene expression values. Each dot in the plot represents each sample/replicate. Principal component 1 (x-axis) explains 68% of the variance in the data and principal component 2 (y-axis) explains 19% of the variance in the data. The dots are coloured according to the treatment condition (red-untreated; blue-treated), and they are shaped according to the types of knockdown (round—shD3#1, triangle—shD3#4, square—siD3#3). **(B, C)** Reactome pathway analysis of A549 cells upon THUMPD3 depletion. Reactome enrichment statistics for top up- and downregulated pathways are plotted and ranked according to adjusted p-value ranging from red (significant) to blue (less significant). **(C)** Western blotting of Laminin B3 levels in A549 cells upon THUMPD3 depletion. Cells were collected 5 days after siRNA transfection for a Western blot analysis with anti-Laminin B3 followed by anti-THUMPD3 antibodies, as indicated. Vinculin levels were used as loading control, as indicated. The asterisk indicates a cross-reaction band. **(E-G)** Volcano plots representing differential gene expression changes from the comparison of shD3#1 vs wt, shD3#4 vs wt, siD3#3 vs wt. The log2 fold change is plotted on the x-axis against -log10 of adjusted p-value plotted on the y-axis. Each point represents a single gene. A single gene with a significant fold change or p-value is represented in green or blue, respectively. When both parameters are significant for a gene, the corresponding dot is displayed in red. Significance cut-off for log2 fold change is 0.585 and for adjusted p-value is 0.05.
(TIF)

S3 Fig Changes in alternative splicing induced by THUMPD3 depletion. **(A-C)** Volcano plots of differential splicing events: intron retention, alternative 3’ splice site, alternative 5’ splice site. The fold change of dPSI is plotted on the x-axis against -log10 of non-changing probability plotted on the y-axis. Each point represents a single transcript. Significance cut-off for p-value is 0.05. **(D)** Targets identified through alternative exon usage analysis were grouped based on their relationship to ECM and CAMs (blue) or links to neurodevelopment (yellow). Transcripts falling into both categories are represented in the green zone; ECM—extracellular matrix, CAM—cell adhesion molecule.
(TIF)

S1 Table RNA-seq analysis of gene expression changes in A549 cells upon THUMPD3 depletion. List of significantly upregulated and downregulated transcripts identified through RNA-seq analysis that change in response to THUMPD3 depletion.
(XLSX)

S2 Table Differential splicing analysis data. List of transcripts identified as transcripts that exhibit alternative splicing events in response to THUMPD3 depletion.
(XLSX)

S3 Table List of reagents and resources. Includes list of qPCR primers, siRNA and shRNA sequences.
(XLSB)

S4 Table Numerical values behind graphs in main and supplementary figures. (XLSX)

S1 File Full western blot pictures. (PDF)
