# Supplementary figures and images for: THUMPD3 regulates alternative splicing of ECM transcripts in human lung cancer cells and promotes proliferation and migration
Source: PLoS One. 2024 Dec 10;19(12):e0314655. doi: 10.1371/journal.pone.0314655 (PMC11630588; doi:10.1371/journal.pone.0314655)

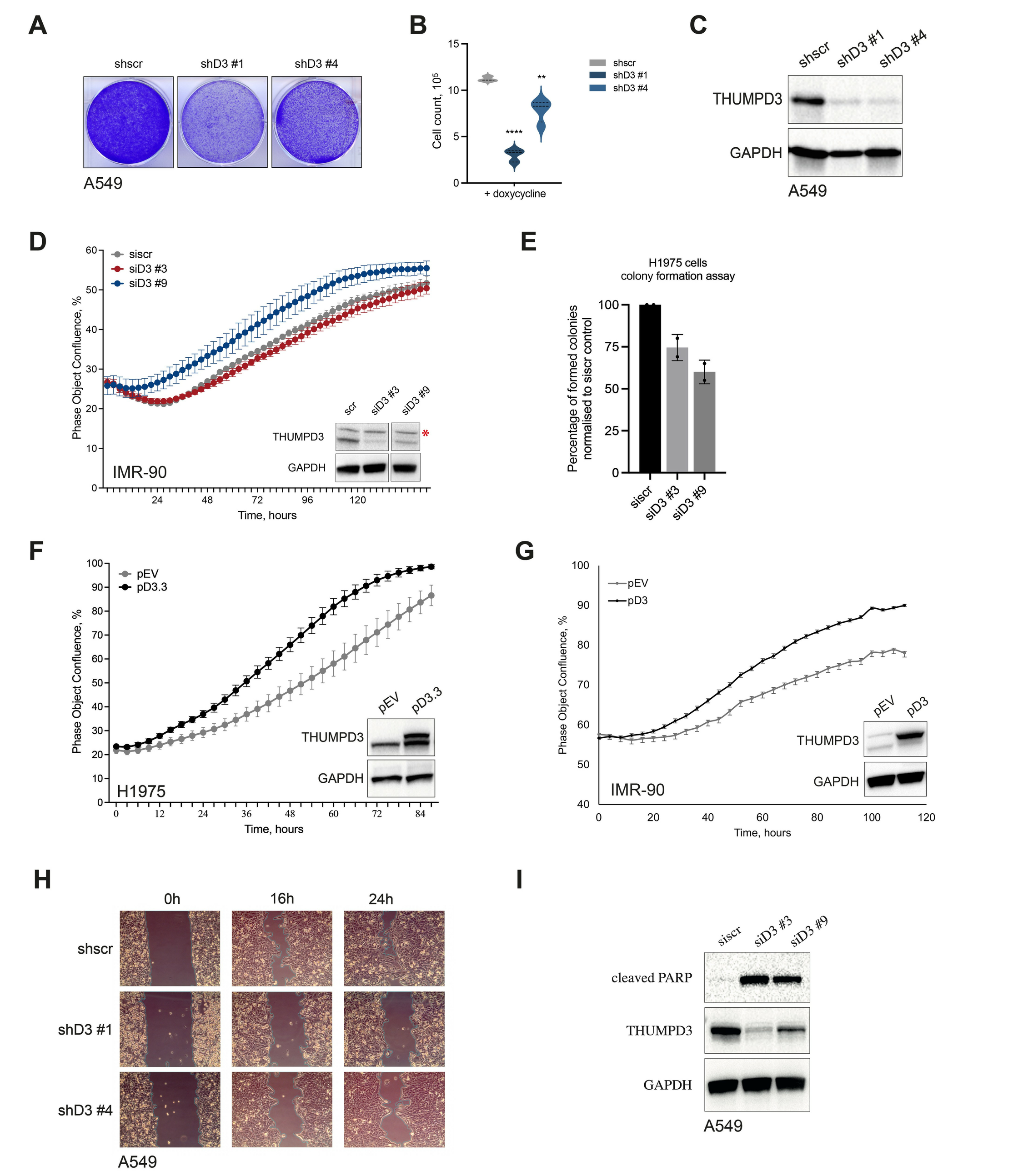

Supplement: S1 Fig — (A) Crystal violet staining of A549 cells with shRNA mediated THUMPD3 depletion (6 days post induction). (B) Effect of shRNA mediated THUMPD3 depletion on cell proliferation assessed by cell counting. The graph represents data from 3 replicates per condition. Statistical analysis was performed using One-Way ANOVA corrected for the comparisons using the Bonferroni method (Alpha: 0.05); ns—P > 0.05, *—P ≤ 0.05, **—P ≤ 0.01, ***—P ≤ 0.001, ****—P ≤ 0.0001. Error bars represent the mean ± SD of 6 independent replicates. (C) Western blotting representing reduction in THUMPD3 level upon shRNA induction. GAPDH was used as a loading control. (D) Live-cell imaging analysis of IMR-90 cell proliferation upon THUMPD3 depletion. Data are represented as the mean of duplicates ± SD. Western blotting was performed to validate THUMPD3 depletion upon siRNA treatment (inset in graph). GAPDH was used as a loading control. The asterisk indicates a cross-reaction band. (E) Quantification (manual) of differences in colony formation in H1975 cells upon THUMPD3 depletion. The bar chart represents percentage of formed colonies normalised to control (mean of 2 experiments ± SD). (F) Live-cell imaging analysis of H1975 cells stably harbouring control empty vector (pEV) or exogenous THUMPD3 expression vector (pD3.3) and transfected with control siRNA (siscr). Following analysis, THUMPD3 levels were assessed by Western blotting (insets in graph). GAPDH was used as a loading control. (G) Live-cell imaging analysis of IMR-90 cells expressing exogenous THUMPD3 (pD3). Following analysis, THUMPD3 levels were assessed by Western blotting (insets in graph). GAPDH was used as a loading control. (H) Wound healing assay upon shRNA mediated THUMPD3 depletion in A549 cells. Representative light-field images of wound healing at indicated time points. Migration fronts are highlighted by blue lines. (I) 200,000 A549 cells were reverse transfected with 2.5 nM of control (siscr) and THUMPD3 (siD3 #3, #9) siRNAs, [file pone.0314655.s001.tif]

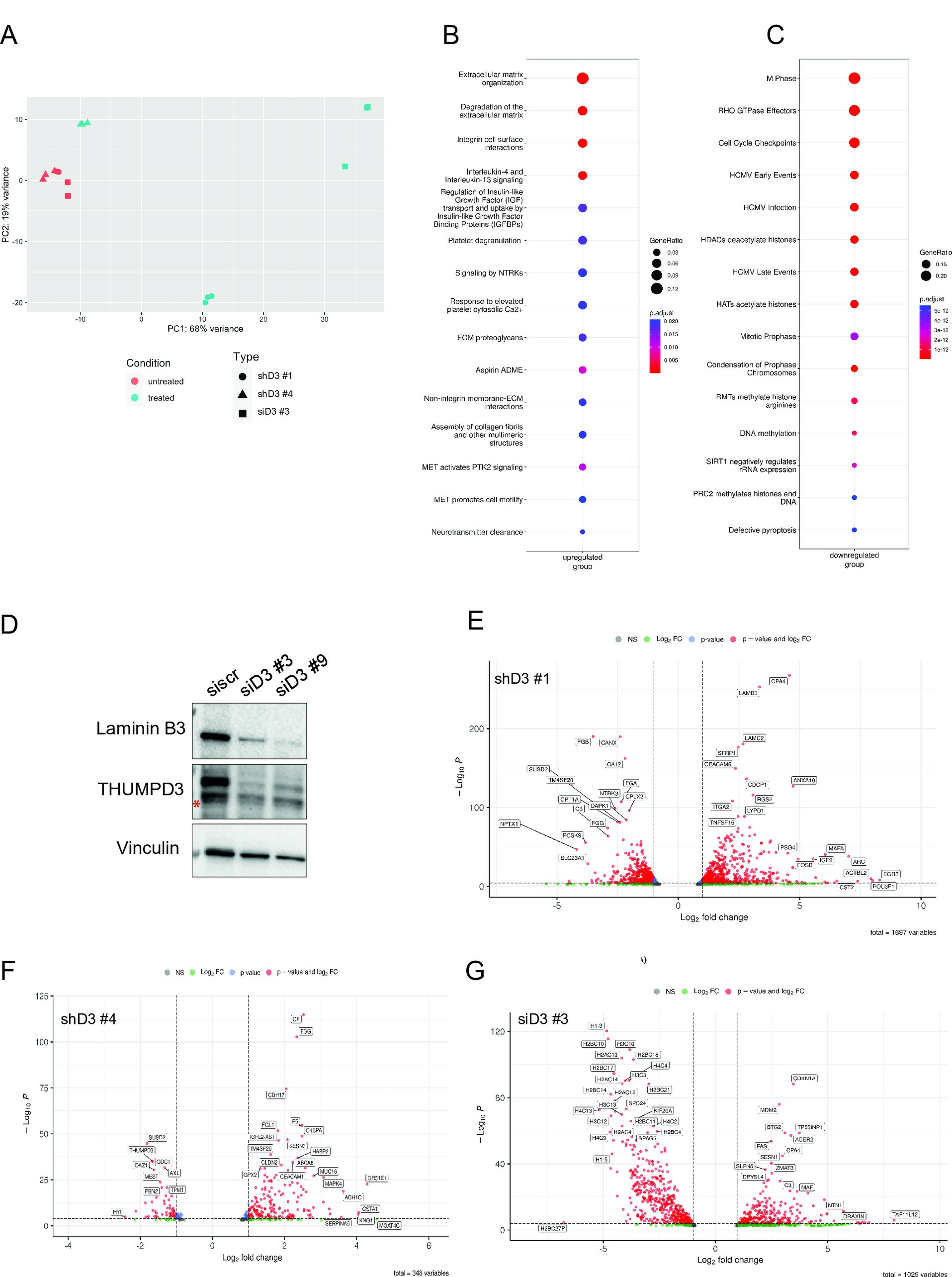

Supplement: S2 Fig — (A) PCA (principal component analysis) based on gene expression values. Each dot in the plot represents each sample/replicate. Principal component 1 (x-axis) explains 68% of the variance in the data and principal component 2 (y-axis) explains 19% of the variance in the data. The dots are coloured according to the treatment condition (red-untreated; blue-treated), and they are shaped according to the types of knockdown (round—shD3#1, triangle—shD3#4, square—siD3#3). (B, C) Reactome pathway analysis of A549 cells upon THUMPD3 depletion. Reactome enrichment statistics for top up- and downregulated pathways are plotted and ranked according to adjusted p-value ranging from red (significant) to blue (less significant). (C) Western blotting of Laminin B3 levels in A549 cells upon THUMPD3 depletion. Cells were collected 5 days after siRNA transfection for a Western blot analysis with anti-Laminin B3 followed by anti-THUMPD3 antibodies, as indicated. Vinculin levels were used as loading control, as indicated. The asterisk indicates a cross-reaction band. (E-G) Volcano plots representing differential gene expression changes from the comparison of shD3#1 vs wt, shD3#4 vs wt, siD3#3 vs wt. The log2 fold change is plotted on the x-axis against -log10 of adjusted p-value plotted on the y-axis. Each point represents a single gene. A single gene with a significant fold change or p-value is represented in green or blue, respectively. When both parameters are significant for a gene, the corresponding dot is displayed in red. Significance cut-off for log2 fold change is 0.585 and for adjusted p-value is 0.05. (TIF) [file pone.0314655.s002.tif]

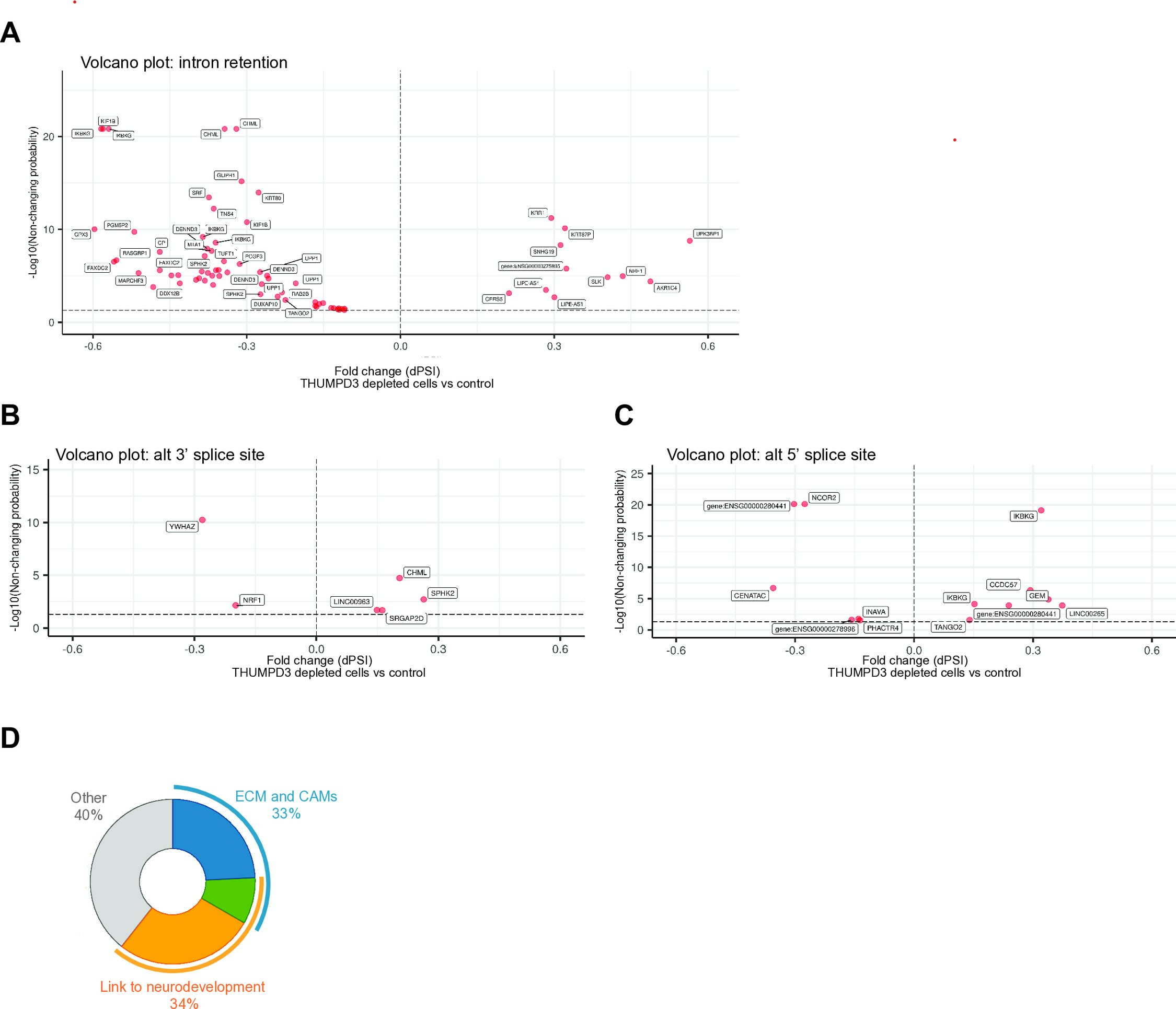

Supplement: S3 Fig — (A-C) Volcano plots of differential splicing events: intron retention, alternative 3’ splice site, alternative 5’ splice site. The fold change of dPSI is plotted on the x-axis against -log10 of non-changing probability plotted on the y-axis. Each point represents a single transcript. Significance cut-off for p-value is 0.05. (D) Targets identified through alternative exon usage analysis were grouped based on their relationship to ECM and CAMs (blue) or links to neurodevelopment (yellow). Transcripts falling into both categories are represented in the green zone; ECM—extracellular matrix, CAM—cell adhesion molecule. (TIF) [file pone.0314655.s003.tif]
