# Supplementary material for: THUMPD3 regulates alternative splicing of ECM transcripts in human lung cancer cells and promotes proliferation and migration
Source: PLoS One. 2024 Dec 10;19(12):e0314655. doi: 10.1371/journal.pone.0314655 (PMC11630588; doi:10.1371/journal.pone.0314655)
Supplement: S1 File — (PDF) [file pone.0314655.s008.pdf]

Figure 1C

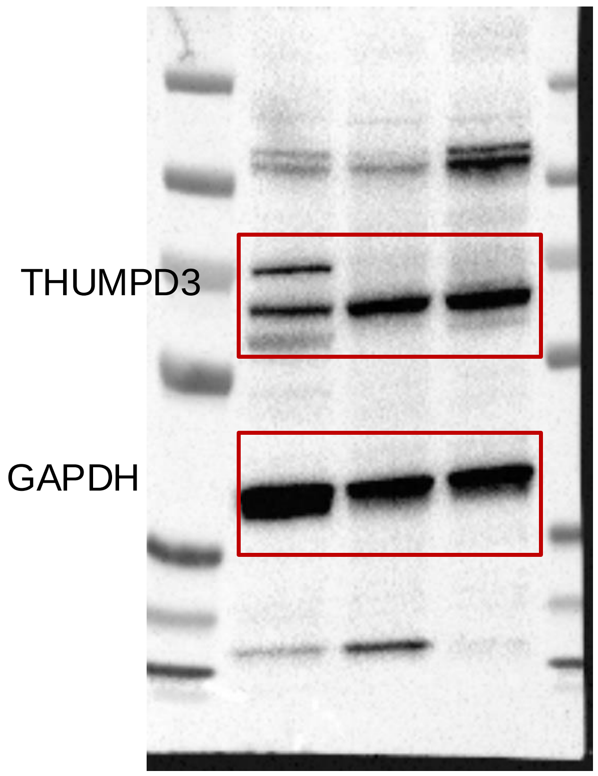

Figure 1E

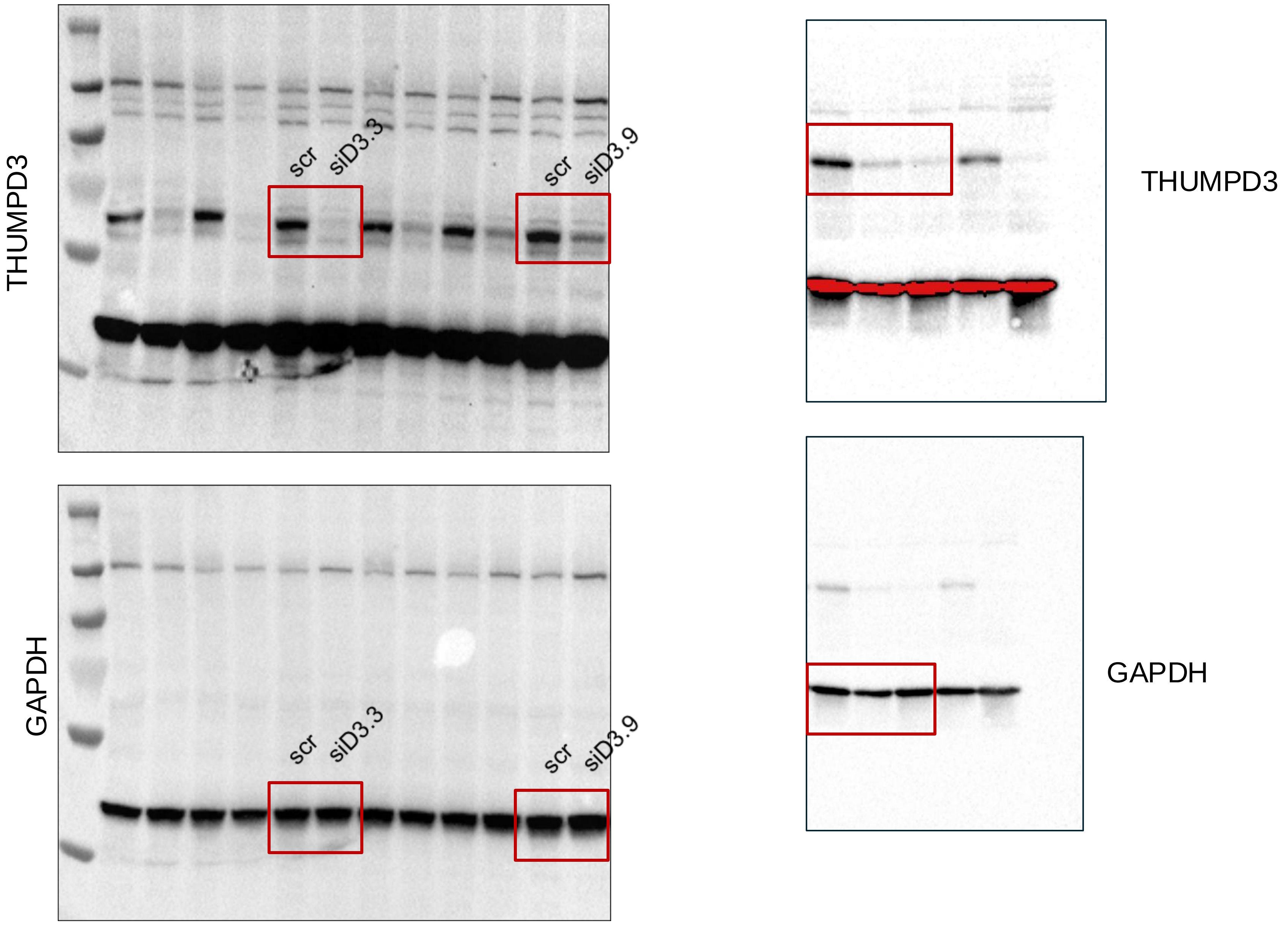

Figure 2B

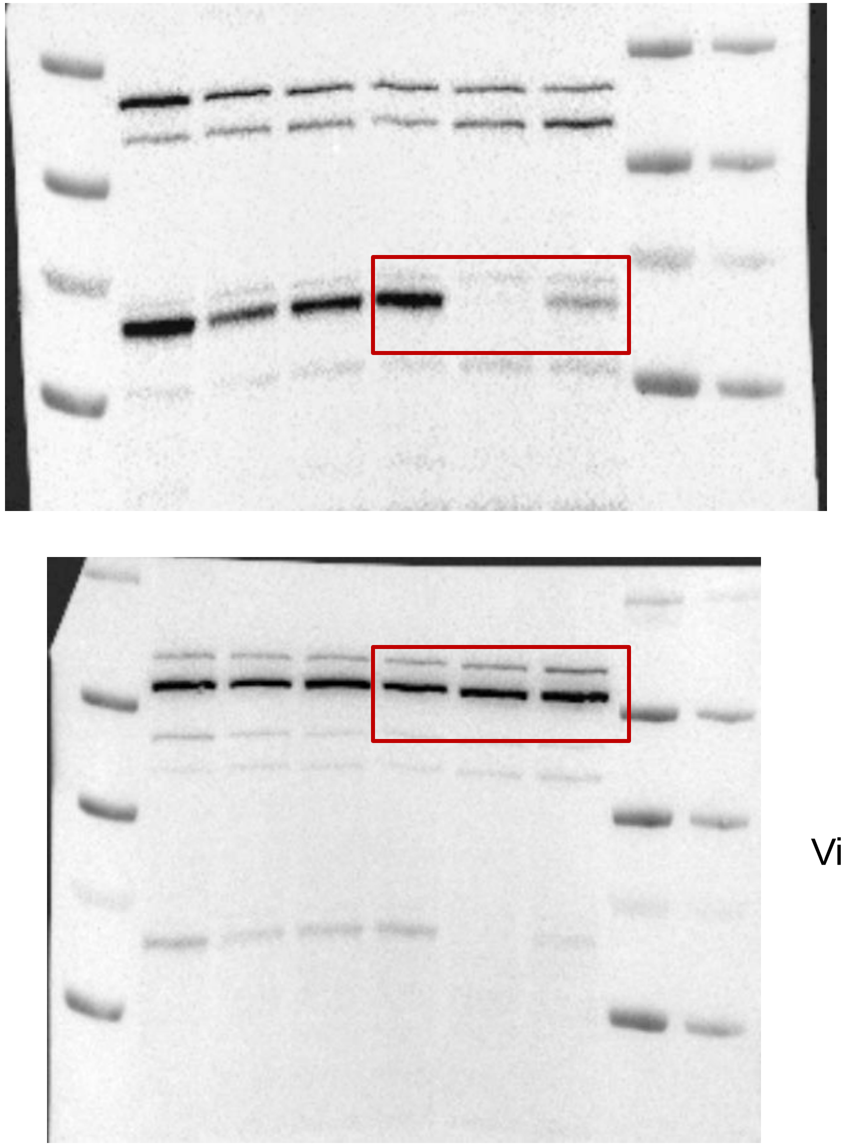

THUMPD3

Vinculin

Figure 2C

Figure S1F

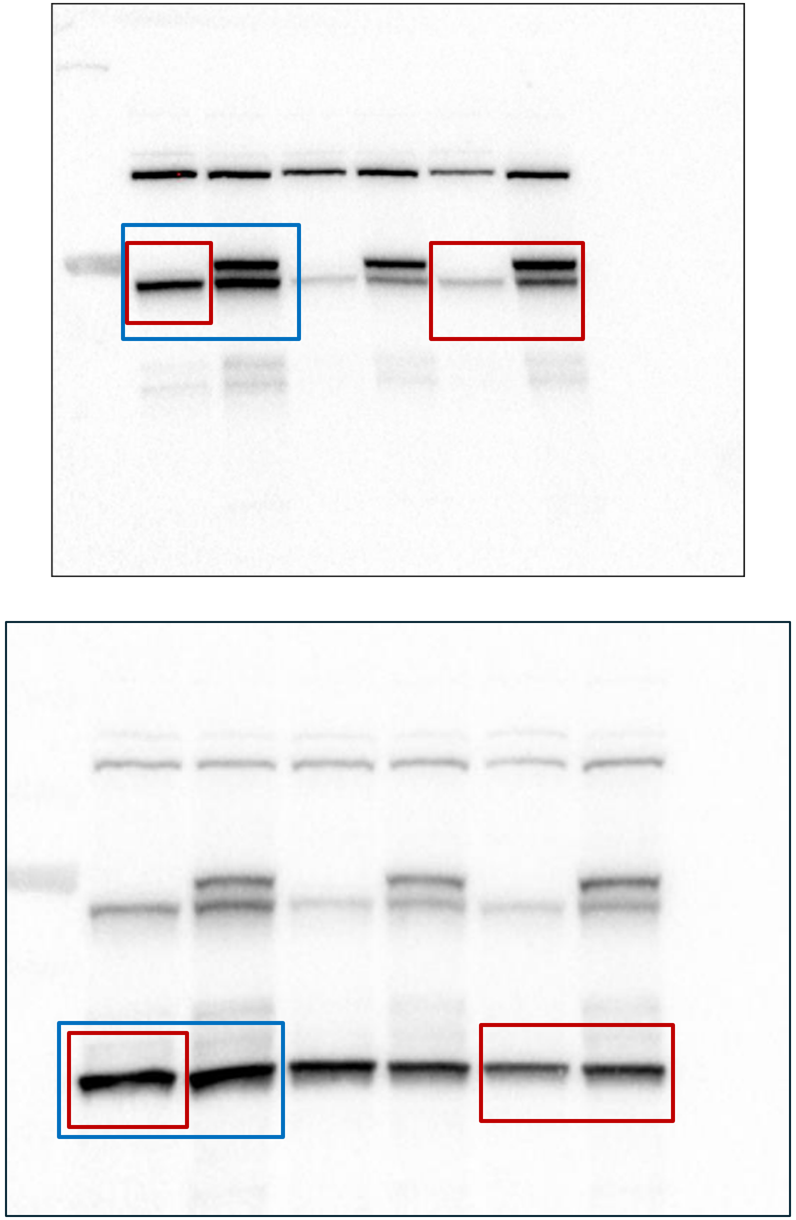

THUMPD3

GAPDH

Figure 3D

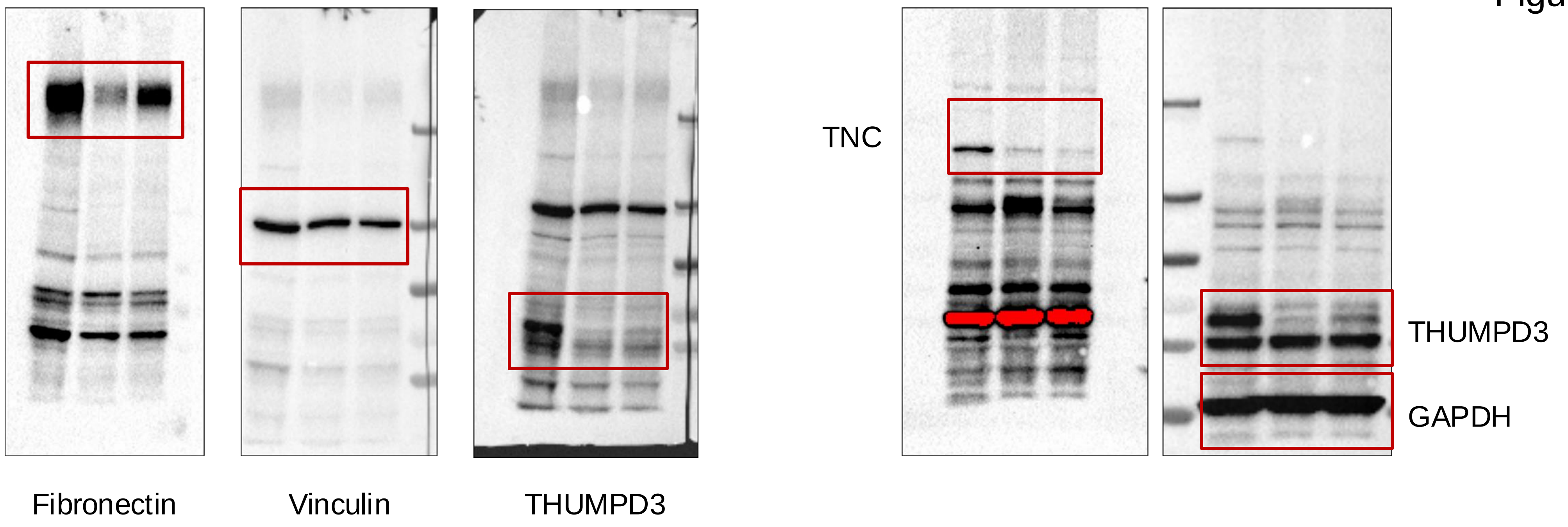

Figure S1C

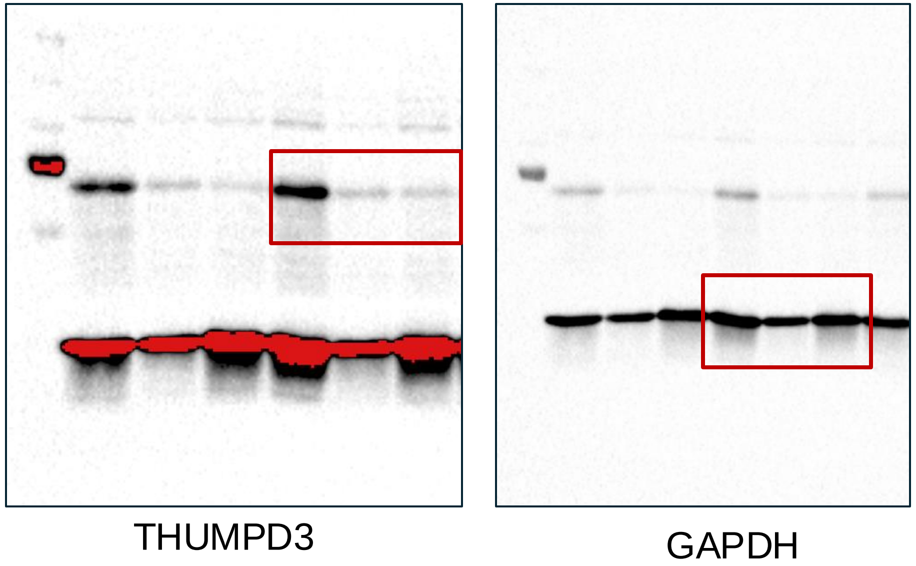

Figure S1D

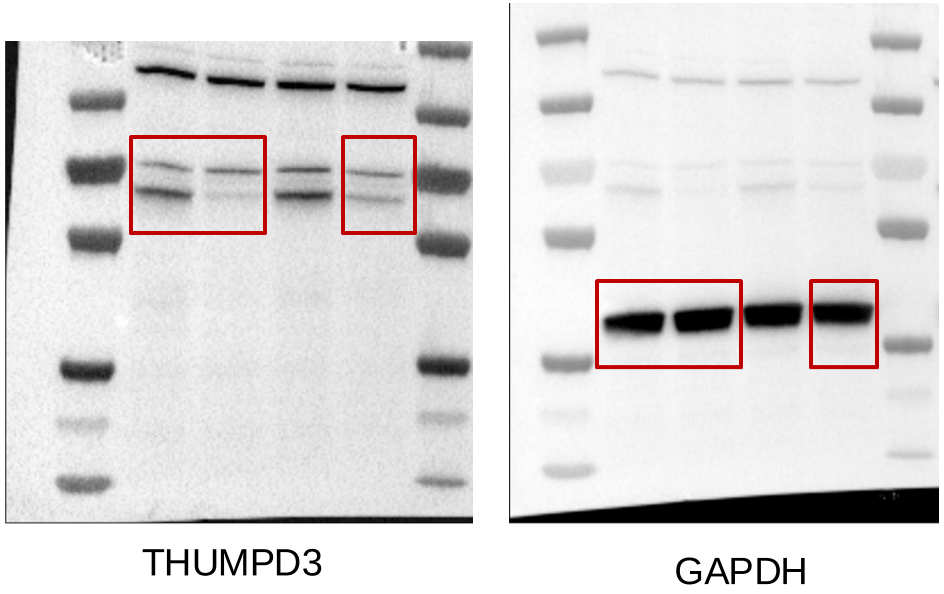

Figure S1G

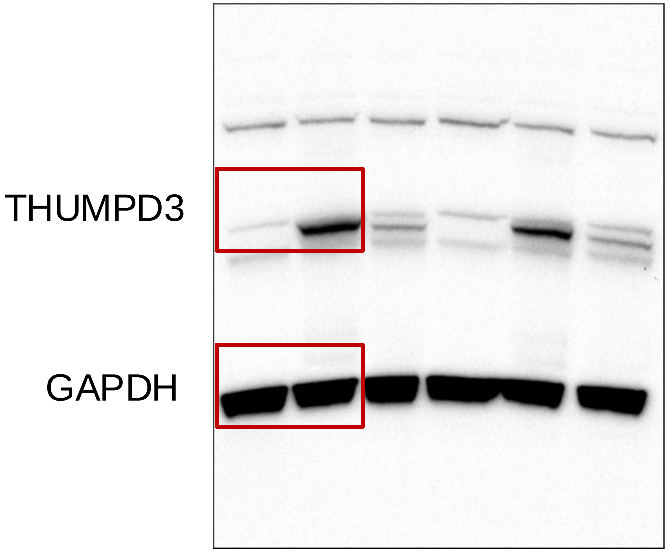

Figure S1I

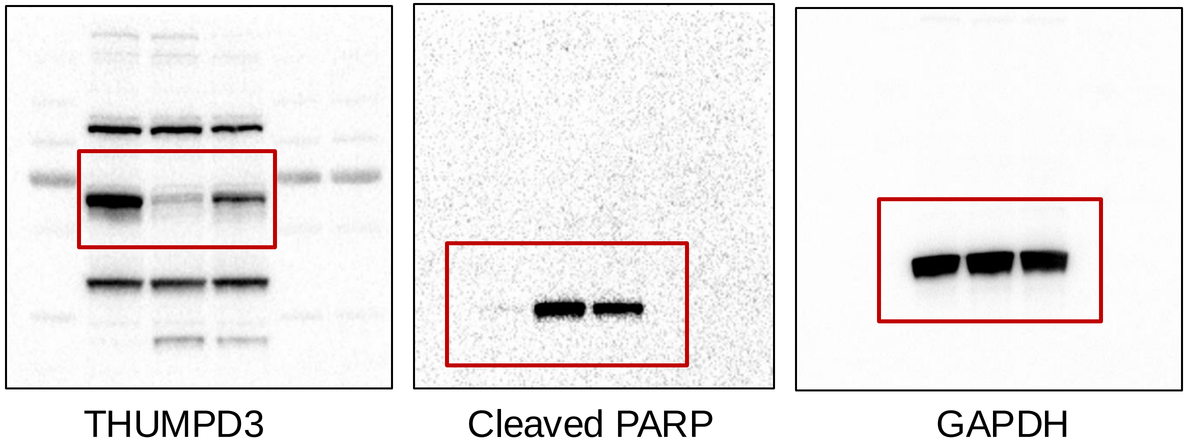

Figure S2D

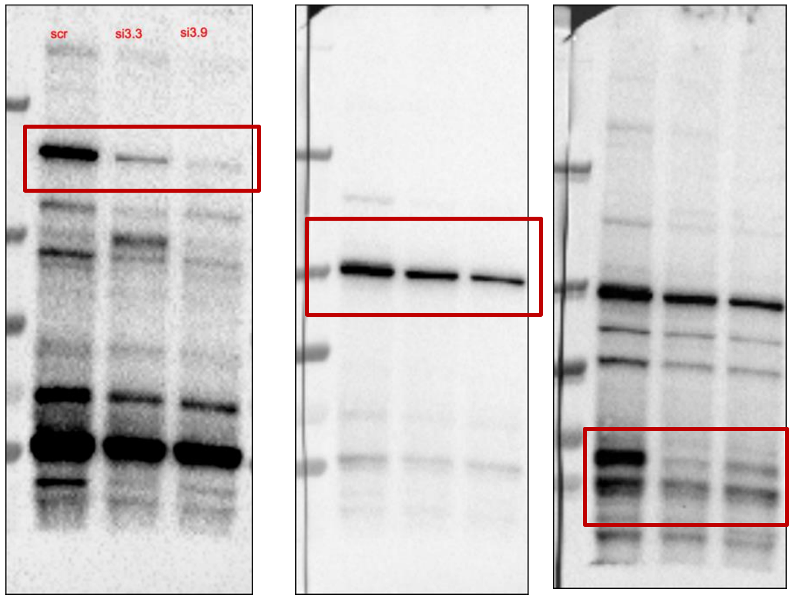

Laminin B3 Vinculin THUMPD3
